# GABA_B_R-Dependent Long-Term Depression at Hippocampal Synapses between CB1-Positive Interneurons and CA1 Pyramidal Cells

**DOI:** 10.3389/fncel.2016.00004

**Published:** 2016-01-28

**Authors:** Dave Jappy, Fliza Valiullina, Andreas Draguhn, Andrei Rozov

**Affiliations:** ^1^OpenLab of Neurobiology, Kazan Federal UniversityKazan, Russia; ^2^Department of Physiology and Pathophysiology, University of HeidelbergHeidelberg, Germany

**Keywords:** inhibition, perisomatic, GABA_A_R, plasticity, interneurons

## Abstract

Activity induced long lasting modifications of synaptic efficacy have been extensively studied in excitatory synapses, however, long term plasticity is also a property of inhibitory synapses. Inhibitory neurons in the hippocampal CA1 region can be subdivided according to the compartment they target on the pyramidal cell. Some interneurons preferentially innervate the perisomatic area and axon hillock of the pyramidal cells while others preferentially target dendritic branches and spines. Another characteristic feature allowing functional classification of interneurons is cell type specific expression of different neurochemical markers and receptors. In the hippocampal CA1 region, nearly 90% of the interneurons expressing cannabinoid type 1 receptors (CB1R) also express cholecystokinin (CCK). Therefore, the functional presence of CB1 receptors can be used for identification of the inhibitory input from CCK positive (CCK+) interneurons to CA1 pyramidal cells. The goal of this study was to explore the nature of long term plasticity at the synapses between interneurons expressing CB1Rs (putative CCK+) and pyramidal neurons in the CA1 region of the hippocampus *in vitro*. We found that theta burst stimulation triggered robust long-term depression (LTD) at this synapse. The locus of LTD induction was postsynaptic and required activation of GABA_B_ receptors. We also showed that LTD at this synaptic connection involves GABA_B_R-dependent suppression of adenylyl cyclase and consequent reduction of PKA activity. In this respect, CB1+ to pyramidal cell synapses differ from the majority of the other hippocampal inhibitory connections where theta burst stimulation results in long-term potentiation.

## Introduction

Long-term plasticity at hippocampal excitatory synapses has been extensively examined. However, much less is known about long lasting changes in GABAergic inhibitory transmission. It is commonly accepted that cooperative action of GABAergic interneurons differentially controls inputs and outputs of hippocampal pyramidal cells (Freund and Buzsaki, 1996; Freund and Gulyas, 1997) and orchestrates coherent oscillations in cortical circuits (Klausberger et al., 2005; Klausberger and Somogyi, 2008). Therefore, long term modulation of efficacy at inhibitory synapses may have a major impact on hippocampal function.

Although the number of studies of long-term plasticity at hippocampal GABAergic synapses is growing, the findings are rather contradictory, reporting both long-term potentiation (LTP) and depression in response to the same induction protocol (Gaiarsa et al., 2002). A possible explanation for this variety is synapse specific recruitment of distinct pre- or postsynaptic signaling cascades. For instance, the same protein kinase (PK) can have different effects on GABA_A_R mediated inhibitory postsynaptic currents (IPSCs) in different cell types (Poisbeau et al., 1999). Within one postsynaptic neuron, the subunit composition of GABA_A_Rs varies between synapses from different presynaptic interneurons. Therefore, the same stimulation paradigm might have distinct effects on different GABAergic synapses.

One of the potential candidates which may be involved in the long lasting changes of GABAergic synaptic efficacy is the GABA_B_R dependent signaling cascade. GABA_B_Rs activate Gαi/o-type G proteins leading to inhibition of adenylyl cyclase, reduced levels of cyclic AMP (cAMP) and lower activity of cAMP- dependent protein kinase A (PKA). This will shift the phosphorylation balance of PKA targets, including GABA_A_Rs, toward the dephosphorylated state (Heuschneider and Schwartz, 1989; Tehrani et al., 1989; Leidenheimer et al., 1991; Moss et al., 1992; Browning et al., 1993; Poisbeau et al., 1999). Depending on subunit composition, PKA phosphorylation can either potentiate or depress GABA_A_R-mediated currents (Feigenspan and Bormann, 1994; McDonald et al., 1998; Vithlani et al., 2011). Involvement of GABA_B_Rs in induction of long-term plasticity via PKA signaling has already been demonstrated for inhibitory synapses (Kawaguchi and Hirano, 2002). In the hippocampus, for instance, GABA_B_R activation upon theta-burst stimulation (TBS) results in LTP of IPSCs in the local inhibitory circuit between stratum lacunosum-moleculare interneurons and CA1 pyramidal cells (Patenaude et al., 2003).

The aim of this study was to explore the possible role of the GABA_B_R in the induction of long-term plasticity at perisomatic synapses between CB1+ putative basket cells and CA1 pyramidal neurons. In the hippocampal CA1 region nearly 90% of the CB1+ interneurons also express cholecystokinin (CCK; Marsicano and Lutz, 1999). The input from CB1+/CCK+ interneurons to pyramidal cells is a powerful inhibitory connection that has unique molecular properties and serves specific functions within the hippocampal network (Armstrong and Soltesz, 2012). *In vivo* data suggest that CB1+/CCK+ basket interneurons are involved in the formation of the theta rhythm (Klausberger and Somogyi, 2008). Postsynaptically, synapses between CB1+/CCK+ cells and pyramidal neurons contain a unique composition of GABA_A_Rs which are mainly assembled from alpha2/beta3 subunits (Nyiri et al., 2001; Kasugai et al., 2010). Therefore, long-term modulation of this connection may have specific mechanisms and important consequences for hippocampal network behavior.

We report that TBS induces robust long-term depression (LTD) at CB1+-Pyr synapses, and induction of this LTD requires activation of GABA_B_Rs, this is in contrast to other hippocampal inhibitory synapses. We show that LTD at this connection involves GABA_B_R-dependent suppression of adenylyl cyclase and consequent reduction of PKA activity.

## Materials and Methods

Transverse hippocampal 300 μm slices were prepared from the brains of 14–21 day-old WT (C57Bl6) mice, killed by cervical dislocation. The slicing chamber contained an oxygenated ice-cold solution (modified from Dugue et al., 2005) composed of (in mM): *K*-Gluconate, 140; *N*-(2-hydroxyethyl) piperazine-*N*′-ethanesulfonic acid (HEPES), 10; Na-Gluconate, 15; ethylene glycol-bis (2-aminoethyl)-*N,N,N′,N*′-tetraacetic acid (EGTA), 0.2; and NaCl, 4 (pH 7.2). Slices were incubated for 30 min at 35°C before being stored at room temperature in artificial CSF (ACSF) containing (in mM): NaCl, 125; NaHCO_3_, 25; KCl, 2.5; NaH_2_PO_4_, 1.25; MgCl_2_, 1; CaCl_2_, 2; and D-glucose, 25; bubbled with 95% O_2_ and 5% CO_2_. During experiments, slices were continuously perfused with the same ACSF. Patch electrodes were pulled from hard borosilicate capillary glass (Sutter Instruments flaming/brown micropipette puller). Electrodes for the postsynaptic pyramidal cells were filled with a solution which consisted of (in mM) Cs-gluconate, 100; CsCl, 40; HEPES, 10; NaCl, 8; MgATP, 4; MgGTP, 0.3; phosphocreatine, 10 (pH 7.3 with CsOH). The solution for the presynaptic interneurons consisted of (in mM) *K*-gluconate, 100; KCl, 40; HEPES, 10; NaCl, 8; MgATP, 4; MgGTP, 0.3; phosphocreatine, 10 (pH 7.3 with KOH).

CA1 pyramidal cells were identified visually using IR-video microscopy. Whole-cell recordings from these neurons were taken at room temperature (23–25°C) in voltage-clamp mode using a HEKA EPC-7 amplifier (List Elektronik) with a sampling rate of 100 μs and filtered at 3 kHz. Cells were held at -70 mV. In paired recordings, presynaptic CB1+ putative basket cells were identified by location and firing pattern. The identity of the presynaptic neuron was further confirmed after finding the connected postsynaptic cell, by asynchronous release evoked by high frequency stimulation (10 action potentials 50 Hz) and the presence of depolarization induced suppression of inhibition.

To extracellularly evoke synaptic currents, glass electrodes filled with ACSF were placed in the stratum pyramidale within ∼50–100 μm of the body of the recorded neuron. Excitatory synaptic transmission was blocked during recordings by the addition of 10 μM NBQX to the perfusion ACSF. A minimal stimulation protocol was used in all experiments with extracellular stimulation. The stimulus intensity was reduced to the lowest amplitude that would cause a response, to reduce the chance of contaminating the response by stimulating many projections. Putative CB1+ interneuron to CA1 pyramidal cell inputs were identified by the presence of pronounced asynchronous release evoked by high frequency stimulation (10 stimuli at 50 Hz) and lasting for at least 100 ms (**Figure 2A**). In LTD experiments, the intersweep interval was 7 s. The theta burst stimulation protocol consisted of four bursts of five stimuli at 50 Hz separated by 200 ms. For LTD induction TBS was repeated 25 times. For analysis, five subsequent responses were averaged and normalized to the mean IPSC amplitude obtained during control recordings prior to TBS or drug application.

The degree of IPSC amplitude changes was calculated as a ratio of the average steady-state current amplitudes before and after TBS or after drug application. Series resistance was monitored, and data from cells in which series resistance varied by >15% during recording were discarded from the analysis.

For statistical analysis, the paired Student’s *t*-test was used, and data are presented as mean ± SD.

## Results

### TBS Induces LTD in CB1+ Interneuron to Pyramidal Cell Connections

**FIGURE 1:**
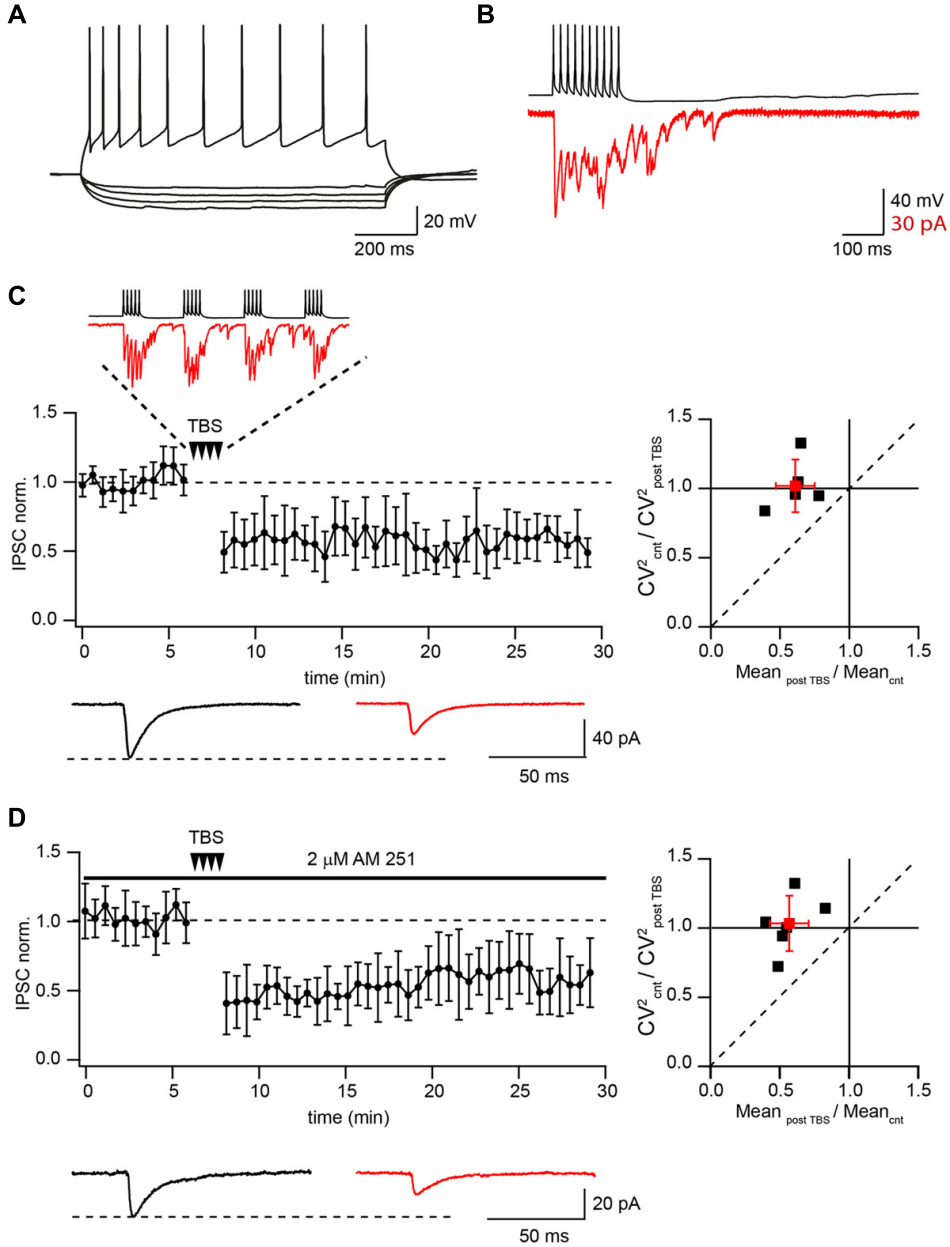
**Theta-burst stimulation (TBS) induces LTD in pairs of connected CB1+ to CA1 pyramidal neurons. (A)** Voltage responses of CB1+ interneuron to hyperpolarizing and depolarizing current injections. **(B)** Inhibitory postsynaptic currents (IPSCs; red) evoked by 50 Hz trains of 10 action potentials (black) in a CB1+ interneuron–pyramidal cell pair. Note profound asynchronous release after the end of presynaptic stimulation. **(C)** TBS triggers long-term depression (LTD) in the pairs of connected presynaptic CB1+ interneurons and postsynaptic CA1 pyramidal cells. The scatter plot (left) shows pooled data from five neurons. The trace above the plot is a typical response to TBS at CB1+ synapses, note profound asynchronous release between AP bursts. Traces underneath are averaged IPSCs prior to (black) and 20 min after (red) TBS. The plot on the right shows result of CV analysis. Black squares represent individual experiments; red square is an average of the data. **(D)** CB_1_R blocker does not affect TBS induced LTD at CB1+ to pyramidal cells connections. The scatter plot (left) shows pooled data from five neuron pairs. Traces underneath are averaged IPSCs prior to (black) and 20 min after (red) TBS. The plot on the right shows result of CV analysis. Black squares represent individual experiments; red square is an average of the data.

It has been shown that TBS triggers LTP in both excitatory and inhibitory hippocampal synapses (Perez et al., 1999; Hoffman et al., 2002; Patenaude et al., 2003; Evstratova et al., 2011). Therefore, we decided to test if this holds true in GABAergic connections between CB1+ interneurons and CA1 pyramidal cells (CB1+ to Pyr). Whole-cell voltage- and current-clamp recordings were performed simultaneously from pairs of connected neurons. Presynaptic interneurons were identified by morphology, location, characteristic firing pattern, and long-lasting asynchronous release evoked by a 50 Hz train of 10 action potentials (**Figures 1A,B**; Hefft and Jonas, 2005). It has been shown that both perisomatic and dendritic targeting CB1+/CCK+ interneurons exhibit a regularly spiking firing pattern but differ in their response to hyperpolarizing current injections (Cope et al., 2002; Evstratova et al., 2011). Schaffer collateral-associated interneurons show a significant hyperpolarization-activated *I*_h_ current and a rebound depolarizing potential often followed by a spike. In contrast to that, CB1+/CCK+ basket cells usually have a small or no *I*_h_ and lack the rebound spike (**Figure 1A**). In this study cells without the *I*_h_ current and the rebound spike were considered as the putative CB1+/CCK+ basket cells.

Presynaptic interneurons were stimulated with a 10 Hz train of two short suprathreshold depolarizing current pulses in current-clamp mode, while IPSCs were recorded in CA1 pyramidal neurons under voltage clamp (*V*_m_ = -70 mV). After obtaining a stable baseline (100 sweeps), the TBS train was repeated 25 times, then at least 150 more sweeps were recorded. In all these experiments TBS resulted in strong LTD with an average IPSC amplitude at a steady state level of 61 ± 17 % relative to control (*n* = 5; *p* < 0.01; **Figure 1C**). Note that during TBS, CB1+ terminals exhibit profound asynchronous release. Paired pulse ratio (PPR) measured before and after TBS did not change significantly (1.16 ± 0.19 vs. 1.17 ± 0.15; *p* > 0.05; *n* = 5). We also analyzed the coefficient of variation (CV; Faber and Korn, 1991) of IPSC amplitudes. The average CV^2^ ratio was close to 1 (1.02 ± 0.19, *n* = 5, **Figure 1C**) pointing toward a postsynaptic locus of LTD induction.

It has been shown that activation of CB_1_Rs can lead to LTD induction at GABAergic and glutamatergic synapses (Robbe et al., 2002; Adermark and Lovinger, 2009; Adermark et al., 2009). Therefore we tested whether endocannabinoids were involved in TBS induced LTD. Application of the CB_1_R antagonist AM-251 (2 μM) resulted in IPSC enhancement (243 ± 65% relative to control; *n* = 6) and PPR reduction due to relief from CB_1_R-dependent tonic suppression of synaptic release (Ali and Todorova, 2010). However, TBS still caused robust LTD even in the presence of AM-251. Relative to control, the averaged IPSC amplitudes were 55 ± 15% (*n* = 6; *p* < 0.01; **Figure 1D**). PPR measured before and after TBS did not change significantly (0.87 ± 0.19 vs. 0.84 ± 0.23; *p* > 0.05; *n* = 6). The average CV^2^ ratio was close to 1 (1.05 ± 0.2, *n* = 6, **Figure 1D**).

### Functional and Pharmacological Identification of Putative CB1+ Interneuron to Pyramidal Cell Connections

**FIGURE 2:**
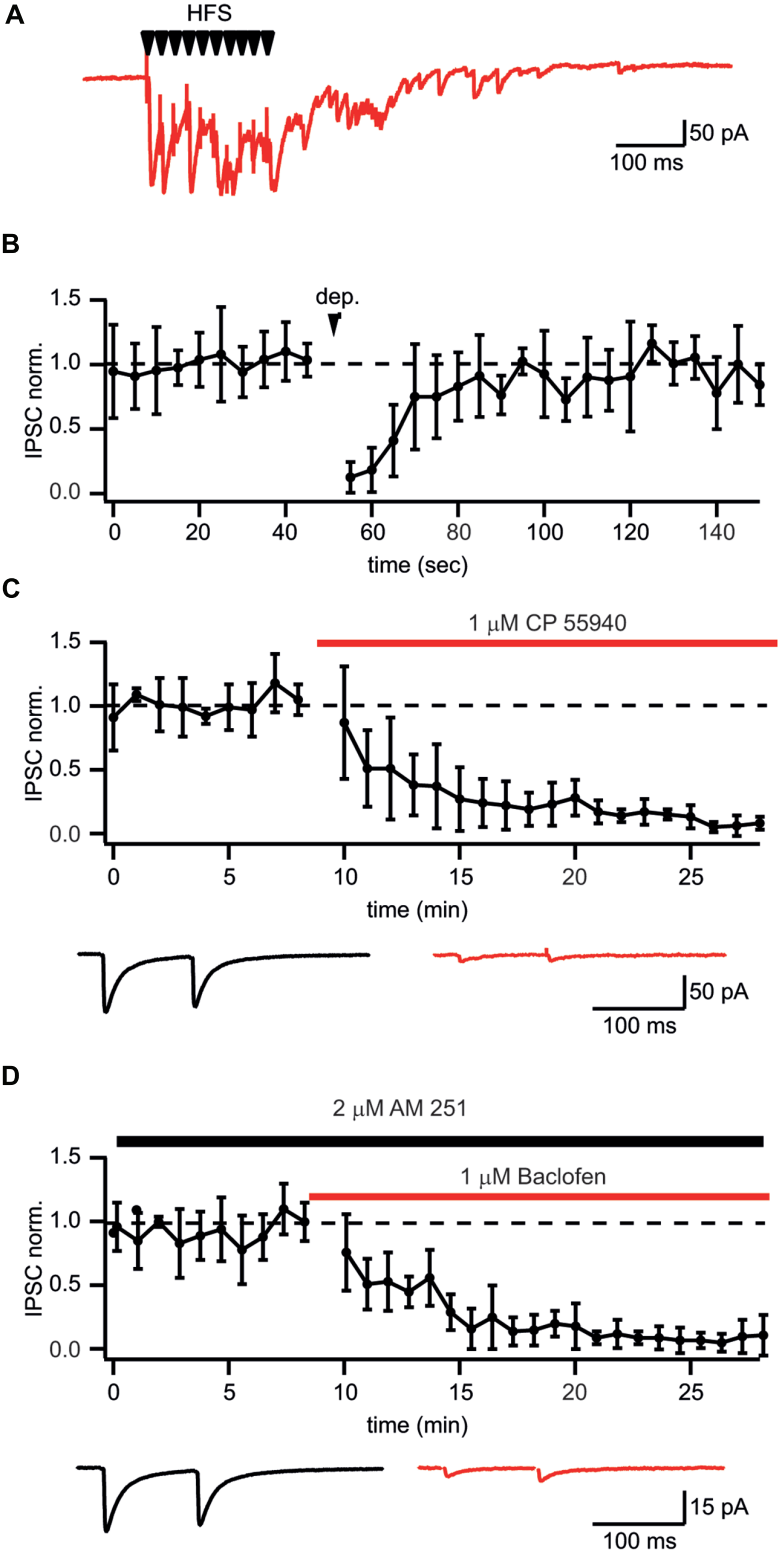
**Identification of putative CB1+ interneuron to CA1 pyramidal cell connections. (A)** Typical postsynaptic response from CB1+ interneurons to-pyramidal cell synapses evoked by high frequency (10 stimuli, 50 Hz) extracellular stimulation. Note long lasting asynchronous release following the stimulation train. **(B)** Depolarization induced suppression of inhibition (DSI) in synapses selected by the presence of asynchronous release. DSI was induced by 5 s depolarization to 0 mV. The scatter plot shows pooled normalized data from five cells. **(C,D)** Effects of the CB_1_R agonist CP55940 **(C)** and GABA_B_R agonist baclofen **(D)** applications on synaptic transmission at synapses with profound asynchronous release. Scatter plots show pooled data from five cells for CP55940 and from six neurons for baclofen. Traces underneath are averaged IPSCs before and after drug application.

It has been shown that prolonged whole-cell dialysis of presynaptic GABAergic neurons can significantly affect vesicle transmitter refilling and, therefore, cause a reduction of postsynaptic IPSCs (Diana and Marty, 2003; Apostolides and Trussell, 2013; Wang et al., 2013). To exclude the “washout effect” on synaptic efficacy we decided to use extracellular stimulation to study plasticity at synapses formed by CB1+ interneurons on CA1 pyramidal cells. One of the difficulties with this stimulation technique is finding reliable conditions and criteria for input specificity. However, as mentioned above, synaptic transmission at these synapses has a number of unique features allowing a relatively easy way to find the proper stimulation location and intensity to ensure activation of a single presynaptic axon. One such feature is robust asynchronous transmitter release following high frequency stimulation. **Figure 2A** shows a typical response to a train of 10 stimuli at 50 Hz when putative CB1+ terminals were stimulated. Another classical way to test for the presence of CB_1_Rs is depolarization induced suppression of inhibition (DSI; Wilson and Nicoll, 2001). To further substantiate our criteria for stimulation settings we tested whether inputs selected by the presence of asynchronous release can undergo DSI. After collecting 25 control sweeps (*Vh* = –70 mV; intersweep interval 5 s.) the postsynaptic CA1 pyramidal neurons were depolarized to 0 mV for 5 s, then an additional 50 sweeps were recorded. In all cases depolarization resulted in strong reduction of IPSC amplitudes (13 ± 12% of control values; *n* = 6; *p* < 0.01; **Figure 2B**). IPSC amplitudes recovered with a time course of about 30 s which is typical for DSI. In an additional set of experiments we showed the expression of CB_1_Rs on asynchronously releasing terminals by testing their sensitivity to the synthetic CB_1_R agonist CP55940. Application of 1 μM CP55940 caused a tenfold reduction of IPSC amplitudes (8 ± 5% of control values; *n* = 5; *p* < 0.01; **Figure 2C**). In addition to CB_1_R, CCK+ terminals show strong GABA_B_R expression (Lee and Soltesz, 2011). Indeed, 10 μM of baclofen suppressed amplitudes of IPSCs down to 15 ± 7% relative to control (*n* = 6; *p* < 0.01; **Figure 2D**). Thus, asynchronous release together with profound DSI is a reliable indicator of CB1+ to CA1 pyramidal neuron input even with extracellular stimulation. The possible level of unspecific stimulation–responses is less than 10% (see **Figure 2C**).

**FIGURE 3:**
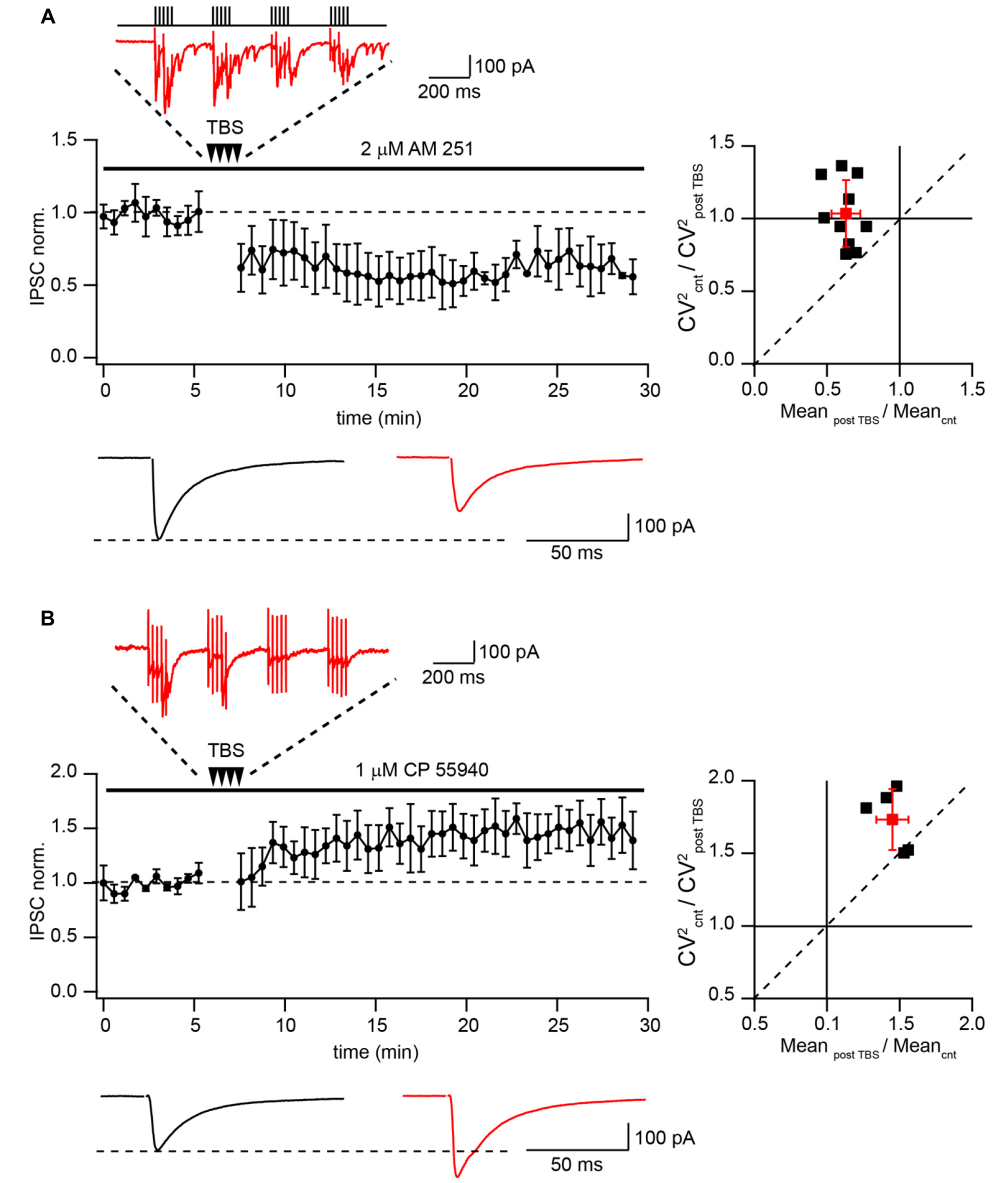
**Differential TBS-induced plasticity at CB1+ and CB1- synapses. (A)** TBS triggers LTD at CB1+ to pyramidal cell connections. The scatter plot shows pooled data from 10 neurons. The trace above the plot is a typical response to TBS at CB1+ synapses, note profound asynchronous release between AP bursts. Traces underneath are averaged IPSCs prior to (black) and 20 min after (red) TBS. The plot on the right shows result of CV analysis. Black squares represent individual experiments; red square is an average of the data. **(B)** TBS-induced LTP at CB1- synapses. CB1+ connections were blocked by bath application of CP55940. Scatter plots summarizes data from five neurons. The trace above the plot is a typical response to TBS at CB1- synapses, note the absence of asynchronous release. Traces underneath are averaged IPSCs prior to (black) and 20 min after (red) TBS. The plot on the right shows result of CV analysis. Black squares represent individual experiments; red square is an average of the data.

Finally we tested whether TBS can produce LTD of extracellularly evoked IPSCs at putative CB1+ to pyramidal cell synapses. A possible contribution of endocannabinoid signaling cascades was excluded by application of the CB_1_R antagonist AM-251 (2 μM) throughout all subsequent experiments. Similarly with the data obtained by paired recordings, TBS resulted in strong LTD with an average IPSC amplitude at a steady state level of 65 ± 9% relative to control (*n* = 10; *p* < 0.01; **Figure 3A**). Note that during TBS, extracellularly stimulated CCK+ terminals exhibit profound asynchronous release similar to that observed with paired recordings. PPR measured before and after TBS did not change significantly (0.78 ± 0.12 vs. 0.71 ± 0.09; *p* > 0.05; *n* = 10). The average CV^2^ ratio was close to 1, indicating a postsynaptic origin of the LTD (1.06 ± 0.23, *n* = 10, **Figure 3A**).

This result contradicts previously reported findings showing that TBS potentiates efficacy at hippocampal GABAergic synapses (Perez et al., 1999; Patenaude et al., 2003; Evstratova et al., 2011). To resolve this apparent contradiction we examined the effect of TBS on CB1-negative (CB1-) inhibitory connections. To this end, release from CB1+ terminals was blocked by activation of CB_1_ receptors with 1 μM CP55940. The stimulation location was in the stratum pyramidale, close to the soma of the recorded neuron. However, despite the very similar recording and stimulation conditions, in the presence of CP55940, TBS triggered significant LTP (147 ± 11% relative to control; *n* = 5; *p* < 0.01; **Figure 3B**). TBS failed to trigger asynchronous release confirming a different origin of postsynaptic responses under these conditions. Moreover CV analysis showed that LTP in CB1 negative synapses is presynaptic. The average CV^2^ ratio was 1.75 ± 0.23 (*n* = 5, **Figure 3B**) Thus, TBS-induced LTD at CB1+ synapses represents a unique property of these synapses.

### Role of GABA_B_Rs in TBS Induced LTD at CB1+ Interneuron to Pyramidal Cell Connections

**FIGURE 4:**
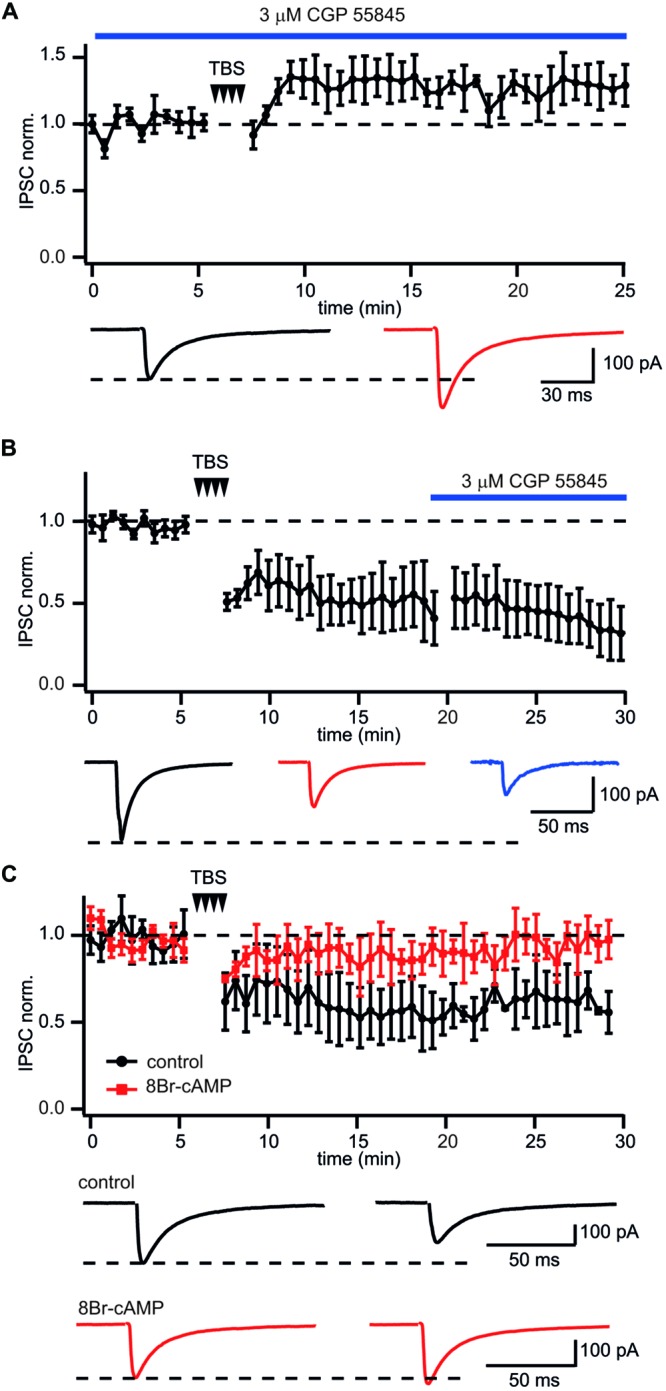
**GABA_B_R involved in LTD induction but not maintenance at CB1+ interneuron to CA1 pyramidal cell connections. (A)** In CB1+ interneuron to pyramidal cell connections blockade of GABA_B_R prior to TBS occludes LTD and triggers potentiation of IPSCs. The scatter plot summarizes data from five neurons. Traces underneath are averaged IPSCs prior to (black) and 20 min after (red) TBS. **(B)** Application of the GABA_B_R antagonist CGP55845 after LTD development does not affect IPSC amplitudes. The scatter plot summarizes data from five neurons. Traces underneath are averaged IPSCs prior to (black), after (red) TBS and in the presence of CGP55845 (blue). **(C)** GABA_B_R dependent suppression of PKA underlies TBS-induced LTD at CB1+ interneuron to pyramidal cell connections. After postsynaptic intracellular administration of the PKA activator 8Br-cAMP TBS fails to induce LTD. The scatter plot compares data obtained with (red squares; *n* = 5) and without (black circles; *n* = 10) 8Br-cAMP in the intracellular solution. Traces underneath are averaged IPSCs prior (left) and after (right) TBS. Responses recorded in the presence of 8Br-cAMP are shown in red.

Next we examined whether GABA_B_R activation is required for LTD induction at CB1–Pyr synapses. To test this, GABA_B_Rs were blocked throughout the experiments by application of the receptor antagonist CGP55845 (3 μM). The presence of CGP55845 did not affect the characteristic properties of CB1+ interneuron to pyramidal cell synapses. However, upon chronic CGP55845 application TBS, instead of triggering LTD of IPSCs, caused significant enhancement of postsynaptic responses. (128 ± 8% relative to control; *n* = 5; *p* < 0.01; **Figure 4A**), strongly suggesting that GABA_B_R activation is involved in the induction of LTD in these synapses.

Long-term depression maintenance may also rely on sustained GABA_B_R activity. To assess this, CGP55845 was added to the perfusion ACSF after a steady state level of LTD had been reached. In this case CGP55845 failed to reverse the LTD. In contrast, IPSC suppression slightly increased after addition of the blocker. Relative to control, the averaged IPSC amplitudes were 51 ± 11% and 41 ± 9% before and after CGP55845 application, respectively; (*n* = 5; **Figure 4B**). Taken together our data suggest that activation of GABA_B_Rs is essential for LTD induction but not for its maintenance.

### LTD at CB1+ Interneuron to Pyramidal Cell Connections Results from GABA_B_R Dependent Suppression of PKA

GABA_B_R receptors are G_i/o_ coupled and have a well-documented inhibitory effect on adenylyl cyclase and PKA activity (Gassmann and Bettler, 2012). Therefore, the cAMP/PKA pathway might be involved in GABA_B_R-mediated long-term LTD. To test this notion, 100 μM of the cAMP analog 8-bromocAMP (8-Br-cAMP) was added to the intracellular solution. **Figure 4C** compares the effect of TBS on synaptic efficacy in the presence and absence of 8-Br-cAMP. Sustained activation of PKA totally blocked TBS-induced LTD. The averaged IPSC amplitude was 96 ± 5% of control value (*n* = 7; *p* > 0.05). These data strongly support the postsynaptic origin of LTD and allows us to suggest that GABA_B_R-mediated suppression of PKA underlies long lasting plasticity at these synapses.

## Discussion

In this study we explored the ability of synapses formed by CB1+ interneurons onto CA1 pyramidal cells to undergo long lasting synaptic plasticity in response to TBS. This stimulation protocol was chosen for two reasons. Firstly, since hippocampal CB1+/CCK+ interneurons have been shown to be involved in generation of theta frequency oscillations *in vivo* (Klausberger and Somogyi, 2008), TBS might represent a natural activity mode for these neurons. Secondly, TBS has been used as an induction protocol to study plasticity in numerous other types of inhibitory connections (Perez et al., 1999; Patenaude et al., 2003; Evstratova et al., 2011) which allows us to compare our data with previously published findings. We found that TBS triggers robust LTD at CB1+ interneuron to pyramidal cell connections. The LTD induction is postsynaptic and requires activation of GABA_B_ receptors. Also, we show that LTD at this connection involves GABA_B_R-dependent suppression of adenylyl cyclase and consequent reduction of PKA activity. This contrasts markedly with other hippocampal synapses, where TBS typically induces LTP. Thus, functionally TBS-like activity will selectively suppresses CB1+ synapses and simultaneously promote other GABAergic inputs, which can have a strong modulatory impact on hippocampal network activity patterns.

### Functional Segregation of Inputs Between CB1+ Interneurons and CA1 Pyramidal Cells

We developed an approach allowing reliable extracellular stimulation of these particular synapses. To accomplish this we used the unique features of CB1+ terminals: long lasting asynchronous transmitter release following high frequency stimulation (Hefft and Jonas, 2005), and exclusive expression of CB_1_Rs (Wilson and Nicoll, 2001). We used asynchronous release as an indication of proper positioning of the stimulation pipettes. The CCK+ nature of these inputs was further confirmed by testing sensitivity of release to cannabinoids. We found that all inputs with characteristic asynchronous release undergo robust DSI in response to 5 s depolarization and also could be almost entirely blocked by application of the CB_1_R agonist CP55940. Thus, asynchronous release can be used as a reliable indicator of CB1+ to-pyramidal cell input even with extracellular stimulation.

### Possible Mechanism of GABA_B_R-Dependent LTD in Synapses Formed by CB1+ Interneurons onto CA1 Pyramidal Cells

In contrast to other inhibitory synapses where TBS triggers LTP, at this connection TBS leads to LTD. Depression seems to be specific for this type of synapse, since the inputs remaining after blockade of release from CB1+ terminals show potentiation under the same stimulation protocol. However, as with TBS-induced LTP, LTD induction at CB1+ interneuron to pyramidal cell connections also requires activation of GABA_B_Rs. Furthermore, GABA_B_R activity leads to LTD via an inhibitory effect on adenylyl cyclase and PKA activity. Recruitment of the same postsynaptic signaling pathway was suggested for LTP induction at a number of GABAergic synapses. What can explain the contradiction between our data and findings reported by other groups? Suppression of PKA should shift the phosphorylation balance of kinase targets toward the dephosphorylated state. The beta subunits of GABA_A_Rs belong to those targets. Moreover, PKA differentially modulates GABA_A_R subtypes, depending on the identity of the beta subunit (Feigenspan and Bormann, 1994; McDonald et al., 1998; Vithlani et al., 2011). Analysis of recombinant GABA_A_Rs has shown that PKA depresses GABA-activated currents in HEK-293 cells expressing beta1-containing GABA_A_Rs, whereas in beta3 containing channels, potentiation was observed (McDonald et al., 1998). Consequently, the final outcome of a reduction in PKA activity will strongly depend on GABA_A_R composition. This might underlie the differences in TBS-induced plasticity between different inhibitory synapses. Indeed, in contrast to the majority of GABAergic synapses which express mainly alpha1-containing channels, GABA_A_Rs at CB1+/CCK+ terminals contain the alpha2 subunit (Nyiri et al., 2001; Kasugai et al., 2010). It has also been shown that the alpha1 subunit assembles with beta2, while alpha2 forms functional channels together with beta3 (Ramadan et al., 2003). Thus, connections between CCK+ interneurons and CA1 pyramidal cells differ from other inhibitory synapses in both alpha and beta subunit identity. At CB1+ interneuron to pyramidal cell synapses, dephosphorylation of synaptic beta3-containing channels due to GABA_B_R-induced reduction of PKA activity, should lead to suppression of GABA_A_R-mediated currents. Alternatively, blockade of GABA_B_Rs can promote PKA phosphorylation and cause potentiation of postsynaptic responses.

### Possible Functional Implications of Differential Long Term Plasticity at CB+/CCK+ Interneuron to Pyramidal Cell Synapses

Hippocampal CB+/CCK+ interneurons constitute a subclass of perisomatic inhibitory cells. Together with parvalbumin-containing basket cells they control the outputs of pyramidal cells. Besides the fact that both types of interneurons target the same compartment, they also receive glutamatergic excitatory drive from the same sources: feed-forward excitation via Schaffer collaterals and feed-back from CA1 pyramids (Freund and Katona, 2007). Moreover, the firing activity of both types of basket cells is phase-locked to theta oscillations *in vivo* (Klausberger et al., 2005; Klausberger and Somogyi, 2008). The TBS stimulation protocol used in this study mimics the *in vivo* theta rhythm activity that normally occurs during exploratory behaviors and sleep. Our findings suggest that during prolonged theta frequency activity, the strength of inputs from CB1+ interneurons can be selectively reduced, leading to a selective disinhibition of a subset of CA1 pyramidal cells. These activity-dependent changes in hippocampal synaptic transmission may contribute to place cell formation and maintenance (Wilson and McNaughton, 1993). In this respect GABA_B_R dependent LTD might have a similar function to endocannabinoid dependent LTD (Younts et al., 2013). In turn LTP at CB1 negative synapses (putative parvalbumin-containing basket cells) might enhance lateral inhibition and further contrast the activity of place cells. Together, our results reveal a new mechanism for activity-dependent modulation of perisomatic inhibition in the hippocampus, with specificity for a well-defined local microcircuit.

## Author Contributions

DJ: acquisition, analysis, and interpretation of data; drafting of manuscript. FV: acquisition, analysis, and interpretation of data; drafting of manuscript. AD: study conception and design; interpretation of data; drafting of manuscript. AR: study conception and design; acquisition, analysis and interpretation of data; drafting of manuscript; final revision.

## Conflict of Interest Statement

The authors declare that the research was conducted in the absence of any commercial or financial relationships that could be construed as a potential conflict of interest.

## References

[B1] Adermark L. Lovinger D. M. (2009). Frequency-dependent inversion of net striatal output by endocannabinoid-dependent plasticity at different synaptic inputs. *J. Neurosci.* 29 1375–1380. 10.1523/JNEUROSCI.3842-08.2009 19193884 PMC2744205

[B2] Adermark L. Talani G. Lovinger D. M. (2009). Endocannabinoid-dependent plasticity at GABAergic and glutamatergic synapses in the striatum is regulated by synaptic activity. *Eur. J. Neurosci.* 29 32–41. 10.1111/j.1460-9568.2008.06551.x 19120438 PMC2661034

[B3] Ali A. B. Todorova M. (2010). Asynchronous release of GABA via tonic cannabinoid receptor activation at identified interneuron synapses in rat CA1. *Eur. J. Neurosci.* 31 1196–1207. 10.1111/j.1460-9568.2010.07165.x 20345910

[B4] Apostolides P. F. Trussell L. O. (2013). Rapid, activity-independent turnover of vesicular transmitter content at a mixed glycine/GABA synapse. *J. Neurosci.* 33 4768–4781. 10.1523/JNEUROSCI.5555-12.2013 23486948 PMC3639006

[B5] Armstrong C. Soltesz I. (2012). Basket cell dichotomy in microcircuit function. *J. Physiol.* 590 683–694. 10.1113/jphysiol.2011.223669 22199164 PMC3381302

[B6] Browning M. D. Endo S. Smith G. B. Dudek E. M. Olsen R. W. (1993). Phosphorylation of the GABAA receptor by cAMP-dependent protein kinase and by protein kinase C: analysis of the substrate domain. *Neurochem. Res.* 18 95–100. 10.1007/BF00966927 8385279

[B7] Cope D. W. Maccaferri G. Marton L. F. Roberts J. D. Cobden P. M. Somogyi P. (2002). Cholecystokinin-immunopositive basket and Schaffer collateral-associated interneurones target different domains of pyramidal cells in the CA1 area of the rat hippocampus. *Neuroscience* 109 63–80. 10.1016/S0306-4522(01)00440-7 11784700

[B8] Diana M. A. Marty A. (2003). Characterization of depolarization-induced suppression of inhibition using paired interneuron–Purkinje cell recordings. *J. Neurosci.* 23 5906–5918. 12843295 10.1523/JNEUROSCI.23-13-05906.2003 PMC6741228

[B9] Dugue G. P. Dumoulin A. Triller A. Dieudonne S. (2005). Target-dependent use of co-released inhibitory transmitters at central synapses. *J. Neurosci.* 25 6490–6498. 10.1523/JNEUROSCI.1500-05.2005 16014710 PMC6725433

[B10] Evstratova A. Chamberland S. Topolnik L. (2011). Cell type-specific and activity-dependent dynamics of action potential-evoked Ca^2+^ signals in dendrites of hippocampal inhibitory interneurons. *J. Physiol.* 589 1957–1977. 10.1113/jphysiol.2010.204255 21486769 PMC3090597

[B11] Faber D. S. Korn H. (1991). Applicability of the coefficient of variation method for analyzing synaptic plasticity. *Biophys. J.* 60 1288–1294. 10.1016/S0006-3495(91)82162-2 1684726 PMC1260182

[B12] Feigenspan A. Bormann J. (1994). Facilitation of GABAergic signaling in the retina by receptors stimulating adenylate cyclase. *Proc. Natl. Acad. Sci. U.S.A.* 91 10893–10897. 10.1073/pnas.91.23.10893 7971979 PMC45132

[B13] Freund T. F. Buzsaki G. (1996). Interneurons of the hippocampus. *Hippocampus* 6 347–470. 10.1002/(SICI)1098-1063(1996)6:4<347::AID-HIPO1>3.0.CO;2-I 8915675

[B14] Freund T. F. Gulyas A. I. (1997). Inhibitory control of GABAergic interneurons in the hippocampus. *Can. J. Physiol. Pharmacol.* 75 479–487. 10.1139/y97-033 9250381

[B15] Freund T. F. Katona I. (2007). Perisomatic inhibition. *Neuron* 56 33–42. 10.1016/j.neuron.2007.09.012 17920013

[B16] Gaiarsa J. L. Caillard O. Ben-Ari Y. (2002). Long-term plasticity at GABAergic and glycinergic synapses: mechanisms and functional significance. *Trends Neurosci.* 25 564–570. 10.1016/S0166-2236(02)02269-5 12392931

[B17] Gassmann M. Bettler B. (2012). Regulation of neuronal GABA(B) receptor functions by subunit composition. *Nat. Rev. Neurosci.* 13 380–394. 10.1038/nrn3249 22595784

[B18] Hefft S. Jonas P. (2005). Asynchronous GABA release generates long-lasting inhibition at a hippocampal interneuron-principal neuron synapse. *Nat. Neurosci.* 8 1319–1328. 10.1038/nn1542 16158066

[B19] Heuschneider G. Schwartz R. D. (1989). cAMP and forskolin decrease gamma-aminobutyric acid-gated chloride flux in rat brain synaptoneurosomes. *Proc. Natl. Acad. Sci. U.S.A.* 86 2938–2942. 10.1073/pnas.86.8.2938 2468163 PMC287035

[B20] Hoffman D. A. Sprengel R. Sakmann B. (2002). Molecular dissection of hippocampal theta-burst pairing potentiation. *Proc. Natl. Acad. Sci. U.S.A.* 99 7740–7745. 10.1073/pnas.092157999 12032353 PMC124338

[B21] Kasugai Y. Swinny J. D. Roberts J. D. Dalezios Y. Fukazawa Y. Sieghart W. (2010). Quantitative localisation of synaptic and extrasynaptic GABAA receptor subunits on hippocampal pyramidal cells by freeze-fracture replica immunolabelling. *Eur. J. Neurosci.* 32 1868–1888. 10.1111/j.1460-9568.2010.07473.x 21073549 PMC4487817

[B22] Kawaguchi S. Y. Hirano T. (2002). Signaling cascade regulating long-term potentiation of GABA(A) receptor responsiveness in cerebellar Purkinje neurons. *J. Neurosci.* 22 3969–3976. 12019316 10.1523/JNEUROSCI.22-10-03969.2002 PMC6757657

[B23] Klausberger T. Marton L. F. O’Neill J. Huck J. H. Dalezios Y. Fuentealba P. (2005). Complementary roles of cholecystokinin- and parvalbumin-expressing GABAergic neurons in hippocampal network oscillations. *J. Neurosci.* 25 9782–9793. 10.1523/JNEUROSCI.3269-05.2005 16237182 PMC6725722

[B24] Klausberger T. Somogyi P. (2008). Neuronal diversity and temporal dynamics: the unity of hippocampal circuit operations. *Science* 321 53–57. 10.1126/science.1149381 18599766 PMC4487503

[B25] Lee S. H. Soltesz I. (2011). Requirement for CB1 but not GABAB receptors in the cholecystokinin mediated inhibition of GABA release from cholecystokinin expressing basket cells. *J. Physiol.* 589 891–902. 10.1113/jphysiol.2010.198499 21173082 PMC3060368

[B26] Leidenheimer N. J. Browning M. D. Harris R. A. (1991). GABAA receptor phosphorylation: multiple sites, actions and artifacts. *Trends Pharmacol. Sci.* 12 84–87. 10.1016/0165-6147(91)90509-Q 1647063

[B27] Marsicano G. Lutz B. (1999). Expression of the cannabinoid receptor CB1 in distinct neuronal subpopulations in the adult mouse forebrain. *Eur. J. Neurosci.* 11 4213–4225. 10.1046/j.1460-9568.1999.00847.x 10594647

[B28] McDonald B. J. Amato A. Connolly C. N. Benke D. Moss S. J. Smart T. G. (1998). Adjacent phosphorylation sites on GABAA receptor beta subunits determine regulation by cAMP-dependent protein kinase. *Nat. Neurosci.* 1 23–28. 10.1038/223 10195104

[B29] Moss S. J. Smart T. G. Blackstone C. D. Huganir R. L. (1992). Functional modulation of GABAA receptors by cAMP-dependent protein phosphorylation. *Science* 257 661–665. 10.1126/science.1323140 1323140

[B30] Nyiri G. Freund T. F. Somogyi P. (2001). Input-dependent synaptic targeting of alpha(2)-subunit-containing GABA(A) receptors in synapses of hippocampal pyramidal cells of the rat. *Eur. J. Neurosci.* 13 428–442. 10.1046/j.1460-9568.2001.01407.x 11168550

[B31] Patenaude C. Chapman C. A. Bertrand S. Congar P. Lacaille J. C. (2003). GABAB receptor- and metabotropic glutamate receptor-dependent cooperative long-term potentiation of rat hippocampal GABAA synaptic transmission. *J. Physiol.* 553 155–167. 10.1113/jphysiol.2003.049015 12963794 PMC2343476

[B32] Perez Y. Chapman C. A. Woodhall G. Robitaille R. Lacaille J. C. (1999). Differential induction of long-lasting potentiation of inhibitory postsynaptic potentials by theta patterned stimulation versus 100-Hz tetanization in hippocampal pyramidal cells in vitro. *Neuroscience* 90 747–757. 10.1016/S0306-4522(98)00531-4 10218776

[B33] Poisbeau P. Cheney M. C. Browning M. D. Mody I. (1999). Modulation of synaptic GABAA receptor function by PKA and PKC in adult hippocampal neurons. *J. Neurosci.* 19 674–683. 9880588 10.1523/JNEUROSCI.19-02-00674.1999 PMC6782188

[B34] Ramadan E. Fu Z. Losi G. Homanics G. E. Neale J. H. Vicini S. (2003). GABA(A) receptor beta3 subunit deletion decreases alpha2/3 subunits and IPSC duration. *J. Neurophysiol.* 89 128–134. 10.1152/jn.00700.2002 12522165

[B35] Robbe D. Kopf M. Remaury A. Bockaert J. Manzoni O. J. (2002). Endogenous cannabinoids mediate long-term synaptic depression in the nucleus accumbens. *Proc. Natl. Acad. Sci. U.S.A.* 99 8384–8388. 10.1073/pnas.122149199 12060781 PMC123076

[B36] Tehrani M. H. Hablitz J. J. Barnes E. M. Jr. (1989). cAMP increases the rate of GABAA receptor desensitization in chick cortical neurons. *Synapse* 4 126–131. 10.1002/syn.890040206 2551053

[B37] Vithlani M. Terunuma M. Moss S. J. (2011). The dynamic modulation of GABA(A) receptor trafficking and its role in regulating the plasticity of inhibitory synapses. *Physiol. Rev.* 91 1009–1022. 10.1152/physrev.00015.2010 21742794 PMC4382539

[B38] Wang L. Tu P. Bonet L. Aubrey K. R. Supplisson S. (2013). Cytosolic transmitter concentration regulates vesicle cycling at hippocampal GABAergic terminals. *Neuron* 80 143–158. 10.1016/j.neuron.2013.07.021 24094108

[B39] Wilson M. A. McNaughton B. L. (1993). Dynamics of the hippocampal ensemble code for space. *Science* 261 1055–1058. 10.1126/science.8351520 8351520

[B40] Wilson R. I. Nicoll R. A. (2001). Endogenous cannabinoids mediate retrograde signalling at hippocampal synapses. *Nature* 410 588–592. 10.1038/35069076 11279497

[B41] Younts T. J. Chevaleyre V. Castillo P. E. (2013). CA1 pyramidal cell theta-burst firing triggers endocannabinoid-mediated long-term depression at both somatic and dendritic inhibitory synapses. *J. Neurosci.* 33 13743–13757. 10.1523/JNEUROSCI.0817-13.2013 23966696 PMC3755719

